# Use of the Distal Facial Artery (Angular Artery) for Supermicrosurgical Midface Reconstruction

**DOI:** 10.1097/GOX.0000000000001978

**Published:** 2019-02-05

**Authors:** Hidehiko Yoshimatsu, Mitsunobu Harima, Takuya Iida, Mitsunaga Narushima, Ryo Karakawa, Shuichi Nakatsukasa, Takumi Yamamoto, Akitatsu Hayashi

**Affiliations:** From the * Department of Plastic and Reconstructive Surgery, Cancer Institute Hospital of the Japanese Foundation for Cancer Research, Tokyo, Japan; † Department of Plastic and Reconstructive Surgery, Graduate School of Medicine, University of Tokyo, Tokyo, Japan.

## Abstract

**Background:**

In free-flap reconstruction of the midface, options for the recipient artery are quite limited; the superficial temporal artery and the facial artery are the most commonly used arteries. We report our approach for the use of the angular artery (the terminal branch of the facial artery) as the recipient artery in free-flap reconstruction of the midface.

**Methods:**

Nine patients with midface defects underwent free-flap reconstructions using the angular artery as the recipient artery. Identification and marking of the facial artery were performed preoperatively using handheld Doppler ultrasound. The angular artery was located through an incision made on the side of the nose. When present, a vena comitans of the facial artery or any subcutaneous vein in the vicinity of the defect was used as the recipient vein. In other cases, the facial vein in the submandibular region was chosen as the recipient vein, using a vein graft.

**Results:**

The average diameter of the angular artery was 0.9 mm (range, 0.7–1.0 mm). In all cases, arterial anastomosis was performed in an end-to-end fashion, and flaps survived completely. In 4 cases, a vein graft was used to bridge the pedicle vein and the facial vein.

**Conclusions:**

Although supermicrosurgical skills may be required for its anastomosis, the angular artery is an anatomically consistent artery, which is suitable for use as the recipient artery in free-flap reconstruction of the midface. Use of the angular artery as the recipient artery allows shorter flap pedicles and decreases the number of vein grafts necessary.

## Abstract 2

Supplemental Digital Content is available in the text.

## INTRODUCTION

Midface reconstruction is challenging, especially when it involves many anatomical structures.^1^ Local pedicled flaps such as forehead flap have been widely used, but they do not suffice reconstruction of multilayer defects. In addition, additional scarring of the face should be avoided in patients susceptible to hypertrophic scar formation. Free-flap transfer can address these issues, but options for the recipient artery are quite limited; the superficial temporal artery and the facial artery are the most commonly used arteries.^2–5^ Although the angular artery (the terminal branch of the facial artery) is closer to the defect, there have been no comprehensive case series looking into the use of the angular artery as the recipient artery due to its small caliber.

In this article, we report our approach for the use of the angular artery as the recipient artery in free-flap reconstruction of the midface. Its feasibility will be validated through clinical cases.

## PATIENTS AND METHODS

From August of 2011 to April of 2017, under the University of Tokyo Hospital ethics committee–approved protocol, 9 patients with midface defects underwent free-flap reconstructions using the angular artery as the recipient artery. There were 7 male and 2 female patients whose average age was 51.8 years (range, 23–89 years). Patients’ demographic data are given in **Table 1**, Supplemental Digital Content, http://links.lww.com/PRSGO/A989.

### Surgical Techniques

**Fig. 1.**
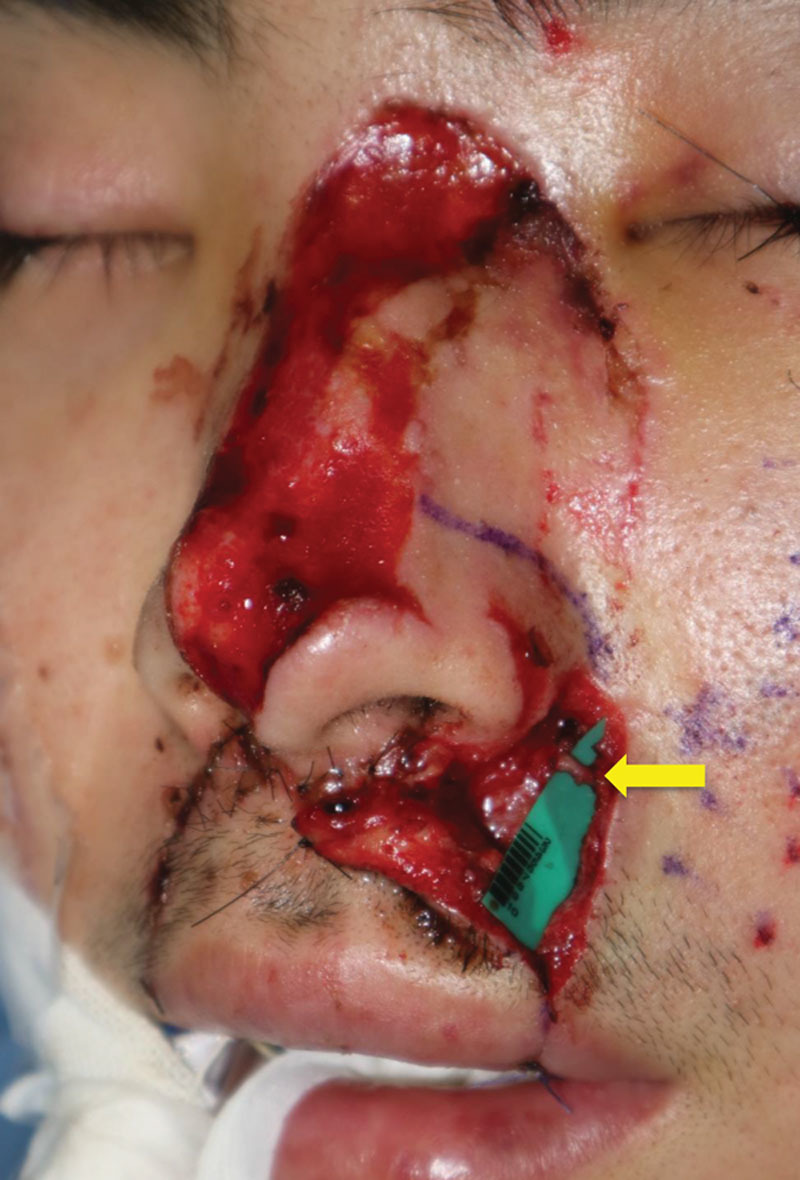
The angular artery with a diameter of 0.8 mm (yellow arrow) was found through an incision made on the side of the nose.

Identification and marking of the facial artery were performed preoperatively using handheld Doppler ultrasound. The angular artery was located through an incision made on the side of the nose (Fig. 1). When present, a vena comitans of the facial artery or any subcutaneous vein in the vicinity of the defect was used as the recipient vein. The direction of venous flow was confirmed using indocyanine green (ICG) angiography in the following approach: 1.0 ml of ICG (Diagnogreen 0.25%; Daiichi Pharmaceutical, Tokyo, Japan) was administered intravenously followed by 10 ml of normal saline. After injection, a fluorescent video recording of the vein was made using an integrated near-infrared illumination system (OPMI Pentero Infrared 800; Carl Zeiss, Oberkochen, Germany). When no appropriate vein could be found around the defect, the facial vein in the submandibular region was chosen as the recipient vein, and a vein graft was procured from the lower leg to bridge the gap. When there were 2 veins in the flap in this setting, a Y-shaped vein graft was harvested. After thoroughly dilating vessels using microsurgical forceps, arterial end-to-end anastomosis was performed in back wall first fashion using 10-0 nylon sutures.

## RESULTS

The causes of the soft tissue defects included resection of malignant tumor (5 patients), trauma (3 patients), and burn (1 patient). The flaps used for reconstruction were superficial circumflex iliac artery perforator flaps in 4 cases, superficial circumflex iliac artery-based lateral femoral cutaneous nerve flaps in 2 cases, a first web space flap in 1 case, a helical rim flap in 1 case, and a posterior auricular artery perforator flap in 1 case. The average diameter of the angular artery was 0.9 mm (range, 0.7–1.0 mm), and the average diameter of the flap artery was 1.0 mm (range, 0.8–1.5 mm). In all cases, arterial anastomosis was performed in an end-to-end fashion. All flaps survived completely. In 2 cases, the flap became congested within 24 hours after the surgery. Reanastomosis of the veins using a new vein graft restored flap status in 1 case, and an additional venous anastomosis using the angular vein alleviated the congestion in the other case. In 4 cases, a vein graft was necessary to bridge the gap between the pedicle vein and the facial vein at the submandibular site. These findings are summarized in **Table 1**, Supplemental Digital Content, http://links.lww.com/PRSGO/A989.

## CASE REPORT

**Fig. 2.**
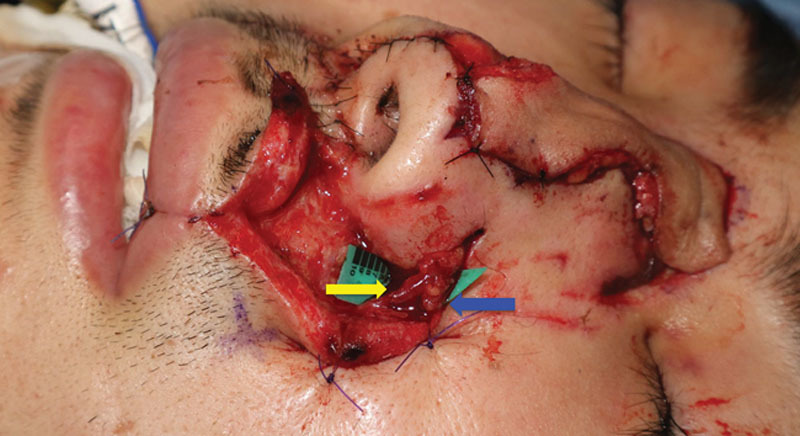
A 5 × 1.2 cm flap was elevated from the posterior region of the left ear, and the artery and the vein of the pedicle were anastomosed to the angular artery (yellow arrow) and the subcutaneous vein (blue arrow), respectively, both in an end-to-end fashion.

**Fig. 3.**
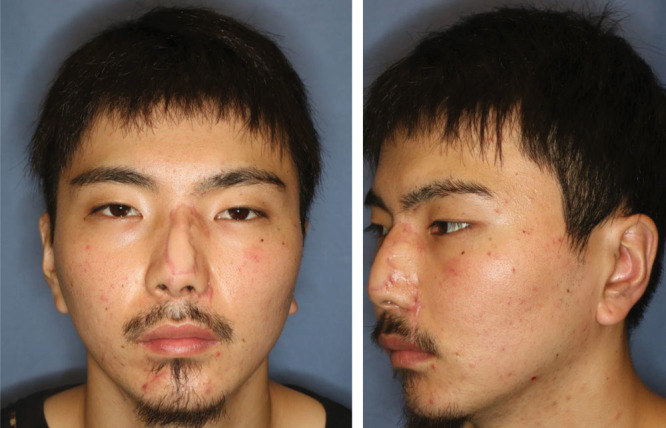
Eight months after the surgery, the flap showed excellent color match and satisfying contour.

A 23-year-old man presented to us with a 5 × 1.2 cm full-thickness defect of the nose after falling from a bicycle. The patient wanted to go back to his work as a salesperson as soon as possible, and was prone to hypertrophic scar formation. Thus, 1-stage reconstruction using a free flap was planned. The angular artery with a diameter of 0.8 mm was found through an incision made on the side of the nose (Fig. 1). A subcutaneous vein with a diameter of 1.0 mm was located in a deeper layer, and the direction of the venous flow was confirmed using ICG angiography (**see Video**, Supplemental Digital Content 1, which displays an intraoperative video demonstrating direction of the venous flow using ICG angiography. The vein, the vessel with a background sheet, flowed from the top to the bottom of the screen. http://links.lww.com/PRSGO/A901). A 5 × 1.2 cm flap was elevated from the posterior region of the left ear, and the artery and the vein of the pedicle were anastomosed to the angular artery and the subcutaneous vein, respectively, both in an end-to-end fashion (Fig. 2). The flap became congested 24 hours after the transfer, but it improved after a venous drainage was added to another subcutaneous recipient vein using a vein graft harvested from the dorsum of the foot. The flap showed excellent color match and satisfying contour 8 months after the reconstruction (Fig. 3).

**Video Graphic 1.**
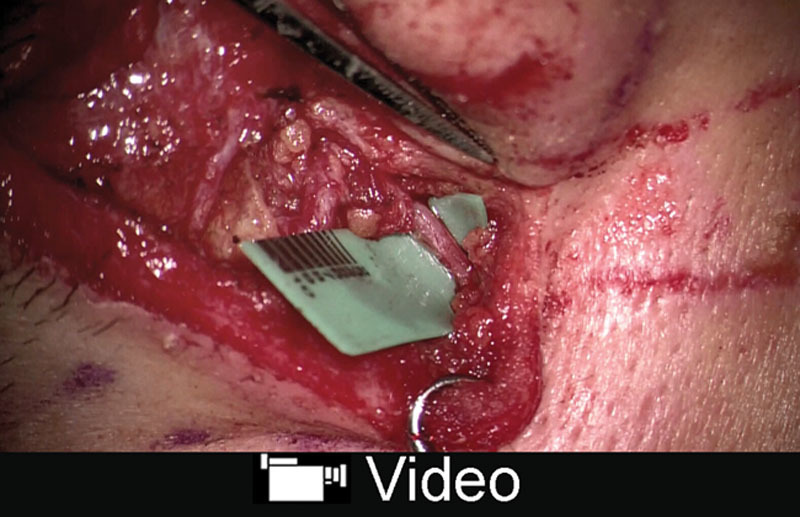
See video, Supplemental Digital Content 1, which displays an intraoperative video demonstrating direction of the venous flow using indocyanine green angiography. The vein, the vessel with a background sheet, flowed from the top to the bottom of the screen. http://links.lww.com/PRSGO/A901.

## DISCUSSION

With the exception of the proximal portion of the facial artery and the superficial temporal artery, few arteries are available for use as the recipient artery in the midface region.^2–5^ The angular artery is in the vicinity of the defect, but probably due to its small size, there have been no comprehensive case series looking into the use of the angular artery as the recipient artery. Zhang et al^5^ reported reconstruction of nasal defects using free-flap transfer involving 66 flaps. Although they described that the facial vessels at the nasolabial fold were first sought, the actual anastomoses were performed on the facial artery and vein in the submandibular region in most cases.^5^ Narushima et al^6^ reported use of the infraorbital artery as the recipient artery; the diameter of the artery was 0.25 mm in their case; however, anastomosis of such small vessels may be impractical for many microsurgeons.

In our case series, the angular artery was found with robust pulsation in all cases, which is an indication for successful free-flap transfer.^7^ To our surprise, the average diameter of the angular artery was 0.9 mm, which was larger than we previously imagined. With many recent reports on supermicrosurgical skills, this size becomes an advantage rather than a disadvantage because it is an ideal match for the pedicle artery of less-invasive perforator flaps such as posterior auricular artery perforator flaps, superficial circumflex iliac artery perforator flaps, or perforator-based anterolateral thigh flaps.^7–9^ The arterial anastomosis can be performed with relative facility in end-to-end fashion.

Once this hurdle of size has been overcome, the use of the angular artery for the recipient artery provides several advantages. First, the closer distance to the defect allows shorter flap pedicles. This can provide safer transfer of flaps with short pedicles, such as posterior auricular flaps, because it precludes the use of vein grafts, and thus decreases the chances of vascular complications. Second, along this line, when the angular artery is used as the recipient artery, the artery will not require a vein graft. In their series with a large number of helical rim flaps, Zhang et al^5^ reported that in about 70% of their 66 flaps, the anastomoses required interposition grafts for both the artery and the vein. Although a vein graft was necessary for venous drainage in 4 out of 9 cases in our series, by reducing the number of vein grafts, one can reduce the chances of postoperative complications.^10^

## CONCLUSIONS

Although supermicrosurgical skills may be required for its anastomosis, the angular artery is an anatomically consistent artery, which is suitable for use as the recipient artery in free-flap reconstruction of the midface. The use of the angular artery as the recipient artery allows shorter flap pedicles and decreases the number of vein grafts necessary.

## Supplementary Material
