# Supplementary material for: Use of the Distal Facial Artery (Angular Artery) for Supermicrosurgical Midface Reconstruction
Source: Plast Reconstr Surg Glob Open. 2019 Feb 5;7(2):e1978. doi: 10.1097/GOX.0000000000001978 (PMC6416107; doi:10.1097/GOX.0000000000001978)
Supplement: Supplementary file 2 [file gox-7-e1978-s002.pdf]

Patients' Demographic Data and Reconstruction Summary

Patient	Age (yr)	Sex	Preoperative diagnosis	Cause of deftect	Diameter of the angular artery (mm)	Type of free flap	Size of skin paddle (cm)	Diameter of pedicle artery (mm)	Recipient vein used	Number and type of vein grafts for venous drainage	Number of vein grafts for arterial anastomosis	Flap complications	Follow-up Length (months)	Flap survival
1	62	Female	Nasal carcinoma	Malignant tumor	1.0	Helical rim flap	3 x 2	1.5	Facial vein	1, lower leg, Y–shaped	1	Congestion	15	Complete
2	23	Male	Skin defect of the nose	Trauma	0.8	Posterior auricular artery perforator flap	4 x1.5	1.2	Subcutaneous veins (2)	1, dorsum of the foot	0	Congestion	8	Complete
3	65	Male	Nasal carcinoma	Malignant tumor	0.7	SCIP flap	10 x 7	0.8	Facial vein	1, lower leg, Y–shaped	0	None	10	Complete
4	41	Male	Nasal carcinoma	Malignant tumor	1.0	SCIP flap	1 x 2	1.0	Facial vein	1, lower leg, Y–shaped	0	None	9	Complete
5	68	Female	Maxillary carcinoma	Malignant tumor	0.8	SCIP flap	19 x 9	0.8	Angular vein	0	0	None	15	Complete
6	69	Male	Nasal carcinoma	Malignant tumor	0.8	SCIP flap	7 x 2	0.8	Angular vein	0	0	None	59	Complete
7	64	Female	Contracture of the lateral commisure of the lips	Burn	1.0	1st web space flap	5 x 3	1.0	Facial vein	0	0	None	10	Complete
8	48	Female	Infraorbital nerve damage	Old zygoma fracture	1.0	SCIP–LFCN flap	0	0.8	Angular vein	0	0	None	13	Complete
9	35	Female	Infraorbital nerve damage	Old zygoma fracture	0.8	SCIP–LFCN flap	0	0.8	Angular vein	0	0	None	8	Complete
Average	51.8				0.9			1.0					16.3	
Range	23–89				0.7–1.0			0.8–1.5					8–59	

SCIP, superficial circumflex iliac artery perforator

LFCN, lateral femoral cutaneous nerve
